# Clinical characteristics of geriatric patients with non-specific chronic low back pain

**DOI:** 10.1038/s41598-022-05352-2

**Published:** 2022-01-25

**Authors:** Yoshihito Sakai, Norimitsu Wakao, Hiroki Matsui, Tsuyoshi Watanabe, Hiroki Iida, Ken Watanabe

**Affiliations:** 1 Department of Orthopaedic Surgery, National Center for Geriatrics and Gerontology, 35 Gengo, Obu, Aichi Prefecture Japan; 2 Department of Bone and Joint Disease, National Center for Geriatrics and Gerontology, Obu, Aichi Prefecture Japan

**Keywords:** Medical research, Pathogenesis

## Abstract

A comprehensive analysis of clinical information in patients with chronic low back pain (CLBP) was performed to clarify the clinical characteristics of geriatric LBP from the perspective of body composition, spinal alignment, and blood findings related to senescence. We enrolled 203 patients with an average age of 79.0 years (77 men and 126 women), with non-specific CLBP as a single-center prospective cohort study, the patients were compared with age- and sex-matched controls without CLBP using a propensity score-matching. We performed laboratory analysis, radiographic evaluations for global spinal parameter and lumbar degeneration, and body composition analysis using whole-body dual-energy X-ray absorptiometry. We observed a higher red blood cell distribution width (RDW) (*p* < 0.001), which is an index of aging, as well as a lower vitamin D level (*p* = 0.002), skeletal muscle mass index (*p* = 0.045) and a higher fat mass (*p* = 0.007) in patients with CLBP. Moreover, patients with geriatric CLBP had significantly lower lumbar lordosis (*p* = 0.024), and higher sagittal vertical axis (*p* = 0.006) was correlated with lower extremity and trunk muscle mass (*p* < 0.001), independent of lumbar degeneration. Geriatric patients with CLBP have sarcopenic fat accumulation and spinal sagittal malalignment with senescent status, such as elevated RDW and hypovitaminosis D.

## Introduction

Low back pain (LBP) is one of the most frequently encountered complaints in clinical setting, and is the most common type of chronic musculoskeletal pain in Japan^1^. The prevalence and associated burden of LBP increase with age^2^; however, research on LBP has primarily focused on youths and adults, and little attention is given to the elderly population^3^. LBP is classified as idiopathic in approximately 85% of patients with LBP^4,5^ considered as non-specific LBP, irrespective of common symptoms and socioeconomic burden^6^. The National Institute for Health and Care Excellence defines non-specific LBP as tension, soreness, and/or stiffness of unknown etiology in the low back region with joint, disc, and connective tissue involvement potentially contributing to symptoms^7^. However, these lumbar spine degenerative changes are highly prevalent with age, and the mechanism whereby they cause LBP remains poorly understood. Alternatively, sagittal alignment of the whole spinal balance to maintain standing postural stability has been recognized for spinal deformity, and related research has been widely conducted^8,9^. Sagittal malalignment has been considered important for the pathophysiology of LBP^10,11^. The relationship between spinal alignment and LBP has been investigated in many clinical studies; however, there are limited and conflicting data on the effects of malalignment on LBP^12^, particularly for geriatric LBP with age-related degeneration of various spinal components. The aging spine, which is characterized by an anterior imbalance of the spine related to the degenerative process requires more energy leading to higher incidence of functional disorders, poor health conditions and earlier mortality^13^. Aging loss of skeletal muscle mass is related to spinal sagittal imbalance^14^. Decreased skeletal muscle mass to support the trunk and changes in the standing posture of the sagittal plane are extremely important as reasons for the high prevalence of LBP in the elderly. Therefore, it is necessary to evaluate the body composition including skeletal muscle and spinal alignment that change with aging in the study of geriatric LBP. Changes in body composition with aging in conclude a decrease in skeletal muscle mass and an increase in fat mass, and the underlying mechanism is that these changes are part of inflammation-based aging^15,16^.

Recent systematic reviews regarding the association between chronic inflammation and non-specific LBP have been published, suggesting that inflammatory cytokines such as TNF-α and IL-6 may be biomarkers of inflammation in the pathogenesis of non-specific LBP^17,18^. Chronic inflammation is well known as a senescence-associated secretory phenotype (SASP), which produces numerous proinflammatory cytokines leading to age-related inflammation (“inflammaging”)^19^. Age-related low muscle mass (sarcopenia) and/or intramuscular fat deposition (sarcopenic obesity), which are associated with geriatric LBP^20,21^, are considered part of systemic inflammation^15,16^. Biobank-based approaches are necessary to elucidate the senescent mechanisms of geriatric LBP; thus, Japanese Cohort Study and Biobank for Non-specific Chronic Pain (J-BINC) has been developed at the National Center for Geriatrics and Gerontology since 2018. This project was established based on clinical data systematically collected by orthopedic specialist (spine and joint surgeon) and biobanking regarding non-specific chronic pain, including LBP, neck pain, and knee pain in the elderly. Regarding chronic LBP (CLBP), most of the research reports describe the potential causes and clinical impacts from the viewpoint of spinal degeneration and malalignment^22^, and there are no studies that address the pathological conditions peculiar to the elderly, such as aging and sarcopenia. We conducted a comprehensive analysis of clinical information in patients with chronic LBP to clarify the clinical characteristics of geriatric CLBP from a perspective of body composition, spinal alignment and blood findings related to senescence.

## Materials and methods

The study protocol was approved by the institutional review board at the National Center for Geriatrics and Gerontology (Approval Number 1229), and carries out in accordance with relevant guidelines and regulations. Written informed consent was obtained from all patients.

### Patients enrollment and eligibility

This observational survey was carried out from January 2018 to April 2020 in our institute from a prospectively collected database in the J-BINC, which is a single-center prospective cohort study recruiting patients from the National Center for Geriatrics and Gerontology to assess whether non-specific chronic pain in geriatric patients shows genetic overlap with clinical findings by the discovery analysis from genome-wide association study. Individuals from both the discovery and replication samples were of Japanese background to limit biases resulting from ethnic disparities, and the clinical database of individuals from the discovery samples was drawn for this study. This cohort was a patient-based study that openly recruited individuals aged ≥ 65 years with non-specific chronic pain lasting for more than 6 months, including LBP, neck pain and knee pain. Non-specific CLBP in this study was defined as follows: (1) LBP with visual analogue scale (VAS) score for LBP ≥ 3; (2) persistent pain localized below the costal margin and above the inferior gluteal folds for more than 6 months; (3) the absence of specific spinal pathology such as infection, tumors, and vertebral fractures on both plain radiographs and lumbar magnetic resonance imaging (MRI); (4) the absence of dominant leg pain caused by radicular and cauda equina disorders; (5) the absence of prominent instability such as spondylolysis, isthmic spondylolisthesis, and degenerative spondylolisthesis more than grade II; (6) no previous lumbar and/or thoracolumbar spine surgery. Degenerated lumbar structures such as the vertebral disc, facet joint, and sacroiliac joint were omitted from the inclusion criteria because available diagnostic procedures for these conditions are inaccurate^22^. In addition to patients who did not meet the above inclusion criteria, we excluded patients who were unable to stand unsupported, could not evaluate VAS due to dementia and did not agree with participation in the present study.

### Age/sex-matched control

The retrospective collection was conducted with data from a prospectively maintained database of Sarcopenia Study for Elderly Patient for patients who underwent whole-body dual-energy X-ray absorptiometry (DXA) and evaluated skeletal muscle mass. Registration in this database requires that whole spine radiograph, lumbar MRI, and blood data be performed within 1 year of DXA. Of 2390 patients (65–100 year, averaged 78.7 years, male 1014 patients, female 1376 patients), 1195 patients excluding lumbar degenerative disease, 683 without complaint of LBP were recruited as control participants.

### Laboratory measurements

Upon enrollment, we collected fasting venous blood samples from patients in the non-specific CLBP and control groups. We recorded complete blood count parameters such as hemoglobin, mean corpuscular volume, white blood cell (WBC) count, lymphocyte count, and red blood cell distribution width (RDW). The RDW is an automated measure of the heterogeneity of red blood cell sizes due to inflammation and senescence of erythropoietic cells in the bone marrow^23^. Additional covariates included albumin, C-reactive protein (CRP), total cholesterol, creatinine, estimated glomerular filtration rate, and 25-hydroxyvitamin D (25-OHD). Serum 25-OHD levels were determined using electrochemiluminescence immunoassay, and 25-OHD concentration was classified as sufficient, insufficient, and deficient for values ≥ 30 ng/mL, 21–29 ng/mL, and ≤ 20 ng/mL, respectively^24^.

### Radiographic evaluation (Fig. 1)

**Figure 1:**
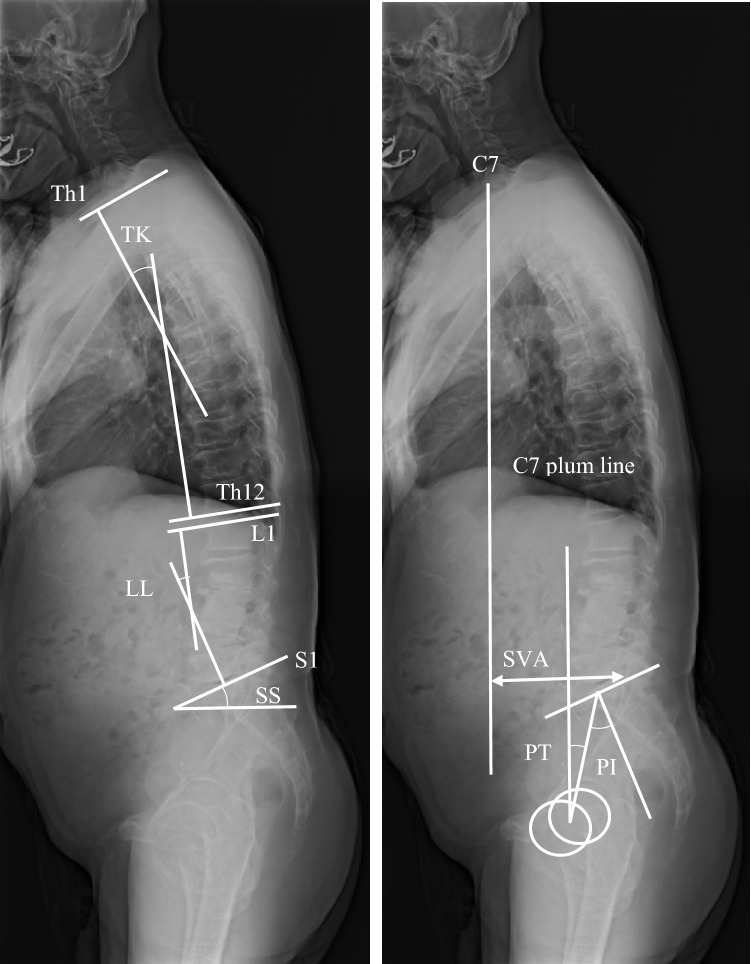
Spinal parameters on standing radiograph. Thoracic kyphosis (TK) and lumbar lordosis (LL) are defined as the angle between the cranial endplate of Th1 and the caudal endplate of Th12, the cranial endplate of L1 and the caudal endplate of L5, respectively. Sacral slope is defined as the angle between the sacral endplate and the horizontal plane. Sagittal vertical axis (SVA) is distance from C7 plumb line from the center of the C7 to the posterior edge of the upper sacral endplate. Pelvic tilt (PT) is defined as the angle is between the line connecting the midpoint of S1 endplate to the center of the femoral head and the vertical line of S1 endplate. Pelvic incidence (PI) is defined as the angle between the line perpendicular to the middle of the cranial sacral endplate and the line joining the middle of the cranial sacral endplate to the center of the femoral head axis.

All patients underwent conventional radiography in the standing position. For lateral films, the patients stood with their knees locked, with feet shoulder-width apart, and looking straight ahead. Measured parameters of interest included coronal Cobb angle between the superior edge of L1 and S1, lumbar lordosis (LL), thoracic kyphosis (TK), S1 slope (SS), sagittal vertical axis (SVA), pelvic tilt (PT), pelvic incidence (PI), the presence of spondylolisthesis (anterior slip > 3 mm), and the lumbar range of motion (ROM) defined as the difference in lumbar lordosis angle between flexion and extension. Spinopelvic mismatch was determined when PI-LL is more than 10°^25^.

### Body composition analysis

Body composition was assessed using DXA (Lunar iDXA, GE-Healthcare, Tokyo, Japan). Osteoporosis was evaluated using the young adult mean on the lumbar spine (L2-4). Sarcopenia was evaluated using the appendicular lean mass derived from the sum of lean mass in the upper and lower extremities with bone mineral content removed, and skeletal muscle mass index (SMI) was calculated using height squared (kg/m^2^)^26^.

### MRI evaluation

Axial T2-weighted slices at L1/2 and L4/5 were obtained to measure the cross-sectional area (CSA) of the lumbar multifidus and erector spinae muscles for the levels of L1/2 and L4/5. Paraspinal muscle CSAs for both the right and left side were added together for each participant. The CSAs were measured using an area calculation software (SYNAPSER, FUJIFILM MEDICAL, Tokyo, Japan). Vertebral endplate and intervertebral disc degeneration were evaluated based on Modic changes^27^ and Pfirrmann classification^28^. End plate and disc degeneration were defined as Modic type I, II, and III (except for type 0), and Pfirrmann grade IV and V, respectively.

### Statistical analysis

We determined that a minimum sample of 394 (197 per group) would be required for a power of 90% to detect a clinically importance between-group difference of 0.35 points (with CLBP vs without CLBP in the elderly patients) in the SMI value. Assumptions for the SMI included a two-sided alpha level of 0.05 and a mean standard deviation of 1.07 points^18^.

Proportions and means with standard deviations (SD) for normally distributed data and median with minimum and maximum values for not normally distributed data were calculated for covariates and demographic information, moreover, categorical variables were expressed as frequencies or percentages. The chi-square or Fisher exact test was used to assess differences in categorical variables, and means were compared using independent t-test and Mann–Whitney U test for normally and non-normally distributed data, respectively. Normality was checked using the Kolmogorov–Smirnov test. To minimize the effects of potential confounding influences of measured covariates in the 2 study groups (CLBP vs. control), a propensity score-matched analysis for age and sex was applied. Finally, patients were matched 1:1 without replacement using a nearest-neighbor approach with caliper restrictions set at 0.2. A propensity score was calculated for each patient using the results of this model, regardless of the statistical significance of the independent variables in the model. The correlation between skeletal muscle mass and spinal sagittal alignment parameters were analyzed using simple linear regression analysis (Pearson correlation coefficient). Statistical analyses were performed using the EZR software (Saitama Medical Center, Jichi Medical University, Saitama, Japan). A *p* value less than 0.05 was considered statistically significant.

### Ethical approval

The device(s)/drug(s) is/are FDA approved or approved by the corresponding national agency for this indication. No benefits in any form have been or will be received from a commercial party related directly or indirectly to the subject of this manuscript. Ethical approval was given by National Center for Geriatrics and Gerontology Ethics Committee.

## Results

**Figure 2:**
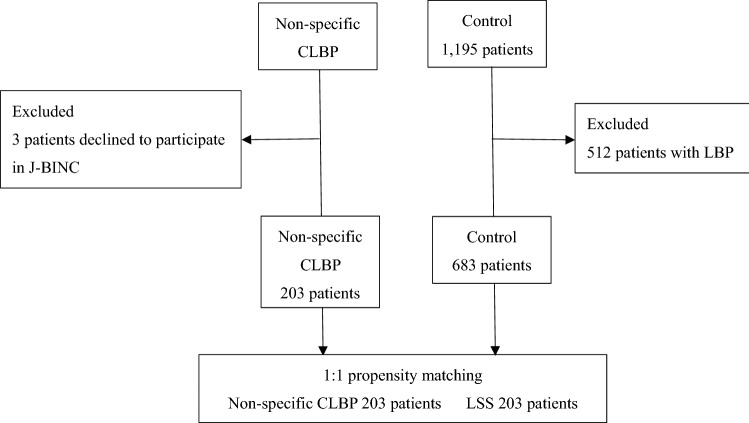
Schematic diagram for patient enrollment of the 2 cohorts. Non-specific CLBP: non-specific chronic low back pain, LSS: lumbar spinal stenosis, J-BINC: Japanese Cohort Study and Biobank for Non-specific Chronic Pain, Control: recruited from database of Sarcopenia Study for Elderly Patient.

We enrolled 203 patients with CLBP with average age of 79.0 ± 6.04 years, comprising 126 (62.1%) women and 77 (37.9%) men. Among patients with CLBP, 203 patients were matched (1:1) for age and sex to control subjects (Fig. 2).

### Laboratory measurements

Demographic and laboratory data of propensity score matching analysis are shown in Tables 1 and 2. There were no significant differences in anthropometry, cytometry, renal function, and nutritional condition. The RDW and the prevalence of elevated RDW were significantly higher in patients in the CLBP group than in those in the control group; however, there were no significant differences in CRP level. Serum 25-OHD levels were significantly lower in patients in the CLBP group than in those in the control group.

**Table 1:** Demographic data.

	CLBP	Control	*p*-value
N	203	203	
Age (year)	79.00 ± 6.04	78.96 ± 5.98	0.934
Sex (M:F)	77:126	77:126	1.000
VAS for LBP	6.61 ± 2.01	1.69 ± 0.55	< 0.001
Height (cm)	153.22 ± 9.41	151.67 ± 9.66	0.103
Weight (kg)	56.42 ± 11.71	55.68 ± 11.53	0.525
BMI	23.92 ± 3.77	24.07 ± 3.63	0.695

*CLBP* chronic low back pain, *VAS* visual analogue scale, *BMI* body mass index.

**Table 2:** Laboratory data.

	CLBP	Control	*p*-value
N	203	203	
Hb (g/dl)	12.96 ± 3.00	12.63 ± 1.51	0.172
Alb (g/dl)	4.07 ± 0.46	4.16 ± 2.14	0.552
T-cho (mg/dl)	192.99 ± 31.60	193.00 ± 35.81	0.998
eGFR (mL/min/1.73m^2^)	64.29 ± 17.59	65.36 ± 16.82	0.532
Cre (mg/dl)	0.79 ± 0.25	0.78 ± 0.26	0.772
WBC (/μL)	5967.98 ± 1632.32	5932.51 ± 1857.80	0.986
Lymphocyte (%)	29.42 ± 9.87	29.97 ± 9.27	0.565
CRP (mg/dl)	0.30 ± 0.68	0.33 ± 1.47	0.805
RDW (%)	Mean 14.01 ± 1.55	Mean 13.43 ± 1.02	< 0.001
	Median 14.00 (12.0–23.0)	Median 13.00 (12.0–16.0)	
Elevated RDW Pts. (%)	32.0	6.90	< 0.001
25OHD (ng/ml)	14.95 ± 7.09	16.89 ± 7.85	0.002
VD deficiency Pts. (%)	80.2	77.7	0.167

*CLBP* chronic low back pain, *Hb* hemoglobin, *Alb* albumin, *T-cho* total cholesterol, *eGFR* estimated glomerular filtration rate, *Cre* creatinine, *WBC* white blood cell, *CRP* C-reactive protein, *RDW* Red cell Distribution Width (cut off≧15.0%), *25-OHD* 25-dihydroxyvitamin D, *VD* vitamin D (< 20 ng/ml = deficiency, < 30 ng/ml insufficiency).

### Body composition analysis

A comparison of body composition is shown in Table 3. Extremity muscle mass, SMI, and trunk muscle CSAs were significantly lower in patients with CLBP than in those with the control. Lower extremity fat mass and body fat ratio were significantly higher in patients with the CLBP than in those with the control.

**Table 3:** Body composition data.

	CLBP	Control	*p*-value
N	203	203	
BMD: L2-4YAM (%)	101.49 ± 26.55	99.68 ± 22.69	0.462
Muscle mass (upper) (g)	3837.64 ± 1170.98	4053.13 ± 1138.93	0.071
Muscle mass (lower) (g)	11,108.69 ± 2608.75	11,734.62 ± 2789.33	0.007
SMI (kg/m^2^)	6.23 ± 0.92	6.43 ± 1.02	0.045
Trunk muscle CSA (L1/2) (mm^2^)	2267.42 ± 804.56	2683.55 ± 836.45	< 0.001
Trunk muscle CSA (L4/5) (mm^2^)	1819.12 ± 770.98	2433.14 ± 715.87	< 0.001
Fat mass (upper) (g)	2086.59 ± 763.62	2089.59 ± 887.54	0.971
Fat mass (lower) (g)	5292.28 ± 1867.88	5142.19 ± 1757.62	0.042
Body Fat (%)	32.17 ± 7.07	29.28 ± 7.48	< 0.001

*CLBP* chronic low back pain, *LSS* lumbar spinal stenosis, *BMD* bone mineral density, *YAM* young mean adult, *SMI* skeletal muscle mass index, *CSA* cross-sectional area.

### MRI evaluation

In reference to lumbar degeneration in patients with CLBP, the frequency of lumbar spondylolisthesis was equal to that in the control and the prevalence of end plate and disc degeneration was lower in patients with CLBP than in the control group. (Table 4).

**Table 4:** Lumbar degeneration.

	CLBP	Control	*p*-value
N	203	203	
Degenerative spondylolisthes (%)	36.0	42.9	0.187
Modic type (0:I:II:III)	101:7:44:51	117:14:36:36	0.050
Modic change (+) (%)	50.7	41.8	0.089
Modic type I (%)	3.7	7.0	0.182
Pfirrmann (II:III:IV:V)	0:38:142:18	0:22:163:13	0.002
Disc degeneration (%)	81.6	90.1	0.015

Modic change (+) and disc degeneration (+) were defined as Modic type I, II, and III except for type 0, and Pfirrmann grade IV and V, respectively.

*CLBP* chronic low back pain, *LSS* lumbar spinal stenosis.

### Radiographic evaluation

**Figure 3:**
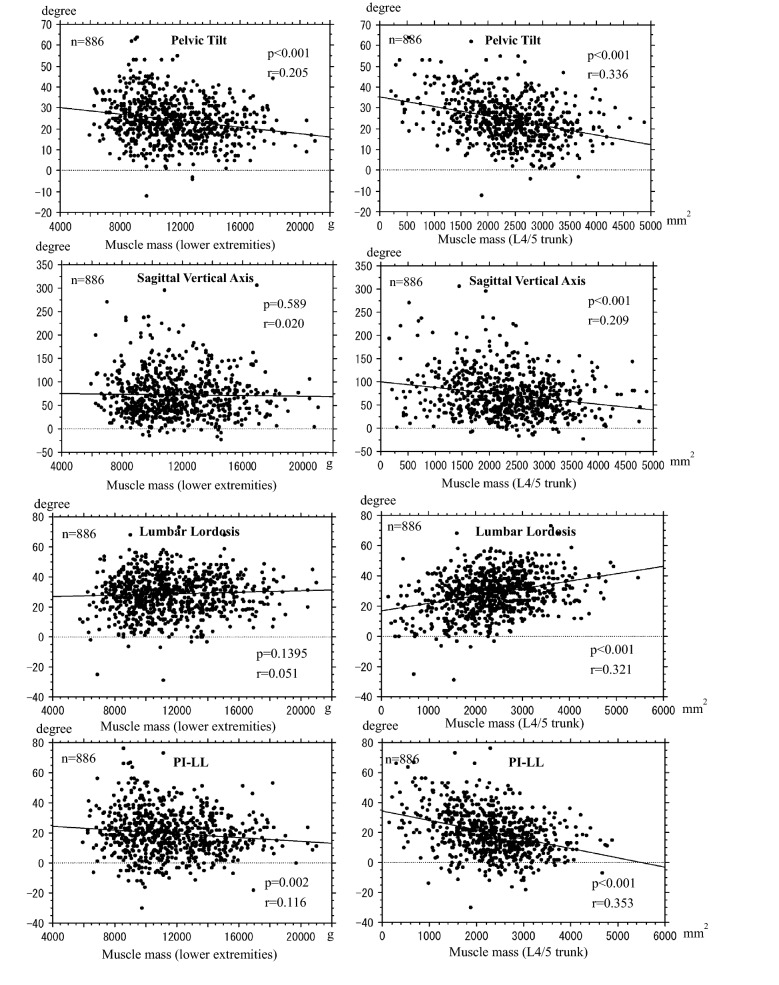
Correlation between skeletal muscle mass and spinal sagittal alignment. Lower muscle mass in both legs and trunk was negatively correlated with PT, whereas only trunk muscle mass was negatively correlated with SVA. Muscle mass in both legs and trunk was negatively correlated with PI-LL; however, trunk muscle mass had stronger correlation with PI-LL compared with lower extremity muscle mass. The total number of plots was obtained by summing 203 cases in the CLBP group and 683 cases in the control group excluding 512 cases with LBP. PT: pelvic tilt, SVA: sagittal vertical axis, LL: lumbar lordosis, PI: pelvic incidence, PI-LL: PI minus LL.

A comparison of spinal sagittal alignment is shown in Table 5. LL was significantly lower in patients with the CLBP than in those with the control, whereas SVA, PT, and PI-LL were significantly higher in patients with the CLBP than in those with the control group. Spinopelvic mismatch was significantly higher in patients with the CLBP group than in those in the control group. Muscle mass in both legs and trunk was negatively correlated with PT, whereas only trunk muscle mass was negatively correlated with SVA. Muscle mass in both legs and trunk was negatively correlated with PI-LL; however, trunk muscle mass was more correlated with PI-LL compared with lower extremity muscle mass. (Fig. 3).

**Table 5:** Spinal sagittal alignment.

	CLBP	Control	*p*-value
N	203	203	
LL (degree)	26.76 ± 13.06	30.12 ± 13.54	0.024
SS (degree)	23.42 ± 9.03	24.30 ± 10.38	0.364
ROM (degree)	25.03 ± 11.35	26.16 ± 10.52	0.301
TK (degree)	36.38 ± 11.53	36.45 ± 11.60	0.954
SVA (mm)	77.87 ± 54.82	61.86 ± 45.49	0.006
PT (degree)	27.65 ± 11.01	22.16 ± 10.52	< 0.001
PI (degree)	51.12 ± 11.78	48.13 ± 12.65	0.019
PI minus LL (degree)	24.20 ± 15.09	19.72 ± 14.80	0.004
Spinopelvic mismatch cases (%)	164 (83.2)	127 (73.0)	0.022

Spinopelvic mismatch was determined as PI-LL ≧ 10°.

*CLBP* chronic low back pain, *LL* lumbar lordosis, *SS* sacral slope, *ROM* range of motion in lumbar spine, *TK* thoracic kyphosis, *SVA* sagittal vertical axis, *PT* pelvic tilt, *PI* pelvic incidence.

## Discussion

In the present study, high RDW related to senescence such as chronic inflammation, oxidative stress, which present in the elderly, were observed in patients with CLBP. Moreover, low lower extremity and trunk muscle mass with high body fat were observed in geriatric patients with CLBP. Previous studies have also reported an association with skeletal muscle mass reduction and LBP in the elderly^18,19^; thus, sarcopenia may have some impact on geriatric LBP development. However, it is unclear how lower extremity muscle mass reduction, which occurs before trunk muscle mass reduction with age^29^, causes LBP in the elderly. Although the relationship between trunk muscle atrophy and LBP has been previously highlighted^30–32^, it has not been concluded whether trunk muscle atrophy is the cause or result of LBP. There is a view that trunk muscle atrophy is caused by disuse, denervation, reflex suppression^33,34^, and there is conflicting evidence for a relationship between the morphological changes in the lumbar muscles and LBP. Age-related skeletal muscle mass reduction originates from type II fibers^35^; therefore, it is known that trunk muscles containing more type I fibers develop sarcopenic changes later than those in the lower extremities^29^. Currently, international guidelines for sarcopenia evaluation, define skeletal muscle mass as the skeletal muscle mass index (SMI), which is dependent on the muscle volume of the extremities^36^. The key to investigating the cause of non-specific LBP in the elderly from the perspective of aging musculature is to proceed with the analysis of geriatric LBP focusing on lower limb skeletal muscle.

Considering the pathophysiological condition of age-related skeletal muscle loss from the molecular biological mechanism of aging, senescence is associated with advanced aging in humans. Senescent cells involving irreversibly proliferative arrest can develop the SASP, consisting of proinflammatory cytokines and extracellular matrix-degrading proteins, which function as deleterious paracrine and systemic mild inflammation^37^. Thus, “inflammaing” is considered as a pervasive feature of aging tissue in age-related diseases^17^. One of the most important organs of locomotor senescence is the skeletal muscle, and sarcopenia, which is an age-related loss of muscle mass, is also considered to be a pathology associated with chronic inflammation mediated by immunosenescence^38^. RDW, which was significantly higher in elderly patients with CLBP in the present study, has been attracting attention as a prognostic factor for various acute and chronic diseases in recent years. This is because it represents the red blood cell size variation and reflects changes in circulatory half-life due to chronic inflammation^39–42^. Elevated RDW is associated with an increased risk of age-related diseases and mortality; moreover, RDW reflects overall inflammation because it is associated with overall and disease-specific mortality risk^41,42^. Veeranna^43^ and Wei^44^ performed comparative analyses of RDW and CRP for mortality prediction in patients with coronary heart disease and infectious endocarditis, respectively. They concluded that RDW, and not CRP, was associated with mortality, independent of traditional risk factors. They also suggested that RDW may be a stronger biomarker for morbidity and mortality. In our study, RDW, and not CRP, was associated with CLBP occurrence in the elderly. This suggests that CLBP may develop in the elderly based on chronic inflammation and RDW may be useful as a biomarker of chronic pain that does not reflect acute inflammation. In addition to chronic inflammation, oxidative stress is another significant mechanism that may explain the elevated RDW. Oxidative damage is an inducer of irreversible cellular senescence mediated by DNA damage, thereby leading to cell survival reduction^45^. Cellular senescence is an irreversible process. Unlike traditional biomarkers of acute inflammation, RDW is a valid indicator of senescence because it is not affected by cases of acute inflammation, such as infectious diseases; more so, it increases over time without large fluctuation, and it has a low reversibility^43,44^. The study results indicate that age-related physical changes in body composition, such as skeletal muscle loss and fat accumulation, are mechanisms of senescence that occur based on chronic inflammation. Thus, senescence might play a role in the development of CLBP in the elderly.

Additionally, recent studies have linked vitamin D, which is known to be effective in preventing falls in the elderly^46^, with chronic pain development^47^. Furthermore, the action of vitamin D is mediated by receptors within muscle cells and bone tissue. Vitamin D is one of the essential elements for the development and maintenance of the musculoskeletal metabolic system, thereby predisposing the patients to sarcopenia^48^. Conversely, vitamin D has anti-inflammatory properties, and pro-inflammatory cytokines are produced during vitamin D deficiency^49^. Vitamin D deficiency causes increased nociceptive skeletal muscle innervation, even before muscle or bone pathology occurs^50^. Moreover, patients with chronic pain and low vitamin D levels are have elevated central hypersensitivity, namely, increased mechanical pain sensitivity and somatic symptom severity^51^. In our study, skeletal muscle mass and 25-OHD levels decreased without bone density loss in patients with geriatric CLBP, which is a noteworthy mechanism of chronic pain mediated by the skeletal muscle system.

The feasibility of application to treatment for geriatric LBP in consideration of the involvement of senescence in the elderly with chronic pain is significant; however, the relationship between inflammation and pain is merely an issue of the pain threshold^52^, and the pathological conditions that trigger the occurrence of LBP are essential. Spinal sagittal alignment, which was significantly different in elderly patients with CLBP in this study, is also one of the most important factors influencing mechanical LBP in elderly patients. Progressive sagittal imbalance is strongly associated with health-related quality of life^8^. Skeletal muscle is important for maintaining sagittal spinal balance; thus, it is conceivable that sagittal imbalance occurs in elderly patients due to age-related muscle mass reduction and/or atrophy other than vertebral fracture. Since patients with vertebral fractures were excluded from this study, the increase in SVA in patients with CLBP was attributed to a decrease in skeletal muscle mass. Although a significant association between skeletal muscle mass reduction and high PT was found in both limbs and trunk, an association between SVA increase and skeletal muscle mass reduction was found only in the trunk. Considering that the decrease in skeletal muscle mass with aging occurs from the lower extremities, it is assumed that the subsequent decrease in trunk muscles accelerates the increase in SVA following pelvic posterior tilt due to skeletal muscle reduction in the extremities. The effect of skeletal muscles on pelvic tilt in our study is consistent with the findings of Hiyama, which demonstrated that pelvic tilt is the sagittal parameter most closely related to skeletal muscle mass in patients with spinal degeneration disease^14^. Studies evaluating LBP and skeletal muscle mass^19,20,53,54^ have reported the effect of skeletal muscle on sagittal spinal balance^14,55,56^; however, our study is the first to analyze the relationship between skeletal muscle reduction and spinal sagittal balance in elderly patients with CLBP. There is no other way than highly invasive surgical treatment to correct spinal deformity in the elderly, and there is no better treatment for sarcopenia. Given the fact that the underlying solution for mechanical LBP is practically challenging, thus it could make sense to seek other treatments to improve geriatric CLBP from an anti-inflammatory and/or anti-senescent perspective.

Our study has a limitation because we used data from heterogeneous patient database of Sarcopenia Study for Elderly Patient for patients who underwent DXA without CLBP as a control. It is unclear whether similar results can be obtained by comparing patients with CLBP and healthy elderly persons. In addition, the cross-sectional study design of our study prevents renders our findings inclusive regarding the role of skeletal muscle mass and spinal alignment in the development of CLBP. Longitudinal investigations on changes in skeletal muscle and spinal parameters in the elderly are needed to clarify the cause of geriatric CLBP.

In conclusion, RDW, which is an index of aging, was high in elderly patients with CLBP. Moreover, geriatric CLBP is associated with vitamin D deficiency, which affects the pain threshold, and triggers CLBP due to the age-related loss of skeletal muscle mass and spinal sagittal malalignment.
